# Authoritarian leadership and cyberloafing: A moderated mediation model of emotional exhaustion and power distance orientation

**DOI:** 10.3389/fpsyg.2022.1010845

**Published:** 2022-10-04

**Authors:** Yajun Zhang, Jingjing Wang, Muhammad Naseer Akhtar, Yongqi Wang

**Affiliations:** ^1^School of Business Administration,Guizhou University of Finance and Economics, Guiyang, China; ^2^Royal Docks School of Business and Law, University of East London, London, United Kingdom; ^3^International College, Guangdong University of Foreign Studies, Guangzhou, China

**Keywords:** authoritarian leadership, cyberloafing, emotional exhaustion, power distance orientation, conservation of resource theory

## Abstract

Drawing upon the conservation of resource theory (COR), this study proposes a moderated mediation model of authoritarian leadership on subordinates’ cyberloafing. Paired samples of 360 employees working in 103 teams from Chinese companies were collected at 2 points in time. The results show that authoritarian leadership positively affects subordinates’ cyberloafing and this relationship is mediated by emotional exhaustion. Furthermore, power distance orientation negatively moderates the relationship between authoritarian leadership and emotional exhaustion and also negatively moderates the indirect effect of authoritarian leadership on subordinates’ cyberloafing through subordinates’ emotional exhaustion. Our findings expand and enrich the literature on authoritarian leadership and cyberloafing and have significant practical implications for managing employees in this digital era.

## Introduction

In the era of the digital economy, organizations increasingly rely on the internet for operational and administrative tasks (Jiang et al., 2020). However, the internet is a double-edged sword. If used properly, it may generate some benefits including faster responsiveness to customer needs, lower operating costs, more effective communication, and better job performance (Lim and Teo, 2005; Liberman et al., 2011; Mercado et al., 2017; Jiang et al., 2020). If mishandled, it may also wreak havoc such as legal disputes, and information security threats (Jiang et al., 2020; Lim et al., 2021). Cyberloafing is a typical form of internet abuse among employees, referring to non-work-related online activities during working hours such as chatting, gambling, playing games, watching online videos, online shopping, and sending private messages (Lim, 2002). Prior research showed that employees spend 40%–60% of their working hours engaging in cyber activities irrelevant to work, which means they spend about 3 h of working time per day or more on the internet (Lim et al., 2021). Cyberloafing is not only common but also concealed, inflicting considerable harm to the development of an organization. Cyberloafing is predicted to result in a yearly financial loss of $4,500 per employee (Lim et al., 2021). In light of this, the identification of its influencing factors and effective control methods is of great practical significance for organizations.

According to an old Chinese proverb, “*soldiers should not move without a general*.” Leaders have a tremendous effect on workers’ cognition, attitude, and behavior as the foundation of any organization. Thus, we consider it imperative to investigate the impact of authoritarian leadership on employee cyberloafing for the following reasons. First, as a result of traditional culture featuring centralization and autocracy, Chinese leaders tend to exhibit paternalistic control, arbitrariness, and authority in managerial practice, resulting in the pervasiveness of authoritarian leadership in local contexts (Chen et al., 2014). Second, the negative impact of a “dark” leadership style on subordinates’ behavior is substantial and far-reaching (Nevicka et al., 2018). As a typical representative of negative leadership styles, authoritarian leadership requires special emphasis and further research scholarship. Thus, the first objective is to examine the relationship between authoritarian leadership and employee cyberloafing.

According to the conservation of resource theory (COR), when individuals deplete their own limited resources, they will try to acquire, maintain, and conserve valuable resources (Hobfoll, 1998). Authoritarian leaders, on the other side, exhibit autocratic, dictatorial, scolding, distancing, and other actions as a cause of stress (Siddique et al., 2020). Employees will lose self-confidence and initiative as a result of these actions, resulting in a loss of individual resources. To cope with authoritarian leaders, employees with insufficient resources have to borrow the resources dedicated to other tasks. Extra resources put in a task frequently do not pay off as expected, resulting in an imbalance and scarcity of individual resources, a sense of resource deprivation, and eventually emotional exhaustion among employees (Hobfoll, 2001). Emotional exhaustion is a mental state caused by excessive psychological and emotional demands that result in a loss of psychological energy and emotional resources. Emotional exhaustion induces a strong sense of self-resource protection and urge to seek additional resources (Hobfoll, 2001). As a result, employees will avoid contact with supervisors and consider cyberloafing as a coping mechanism (Andreassen et al., 2014; Khansa et al., 2017; Farivar and Richardson, 2020), and regain resources through online entertainment activities (Koay et al., 2017; Wu et al., 2020a,b). Therefore, the second research objective is to investigate the mediating role of emotional exhaustion between authoritarian leadership and employee cyberloafing.

In addition, COR theory emphasizes that individual traits to some extent affect the process of acquiring and losing self-resource. This idea aligns well with power distance orientation, which is one of the psychological traits that represent cultural value orientation at an individual level (Kirkman et al., 2009). Power distance orientation also reflects whether individuals understand power-related stimuli as a source of pressure (Peltokorpi and Ramaswami, 2019; Yan et al., 2020). Individual power distance orientation research can help to expose the problem of resource loss caused by authoritarian leadership. The interaction effect may be amplified by the combination of authoritarian leadership and power distance orientation. Specifically, employees with high power distance orientation maintain a social distance from their leaders, abide by the power gap between them and leaders, and do not actively participate in leadership decisions beyond their authority (Lian et al., 2012). Since these employees tend to take the authoritarian behavior of leaders for granted, authoritarian leadership is less likely to produce psychological pressure, resource depletion, and resource imbalance. Thus, authoritarian leadership is less likely to cause emotional exhaustion among employees with high power distance orientation. On the contrary, employees with low power distance orientation wish to participate in decision-making and communicate with leaders. Authoritarian leaders violate the expectation of these employees, aggravate their resource consumption, and so increase the likelihood of exhausting their resources and eliciting negative reactions from them. Therefore, the third research objective is to examine the moderating effect of power distance orientation on the relationship between authoritarian leadership and emotional exhaustion.

In sum, this study intends to investigate the influence of authoritarian leadership on employee cyberloafing based on the conservation of resource theory *via* the mediating role of emotional exhaustion and the moderating role of power distance orientation. Our research makes three contributions to the existing literature. First, this study seeks to add to the current literature on the antecedents of cyberloafing by examining the relationship between authoritarian leadership and employee cyberloafing. Second, this study looked at the role of emotional exhaustion in buffering the relationship between authoritarian leadership and employee cyberloafing. Finally, this study introduced power distance orientation as a moderating variable, which helped to identify the boundary condition of authoritarian leadership causing employees’ emotional exhaustion and cyberloafing.

## Theoretical overview and hypotheses development

### Antecedents of cyberloafing

Scholars have paid close attention to cyberloafing since the term was first proposed in academia. After a careful review of the existing literature, we summarized the influencing factors of cyberloafing into five categories:

1. Demographic variables, including gender, age, educational attainment, and others (Vitak et al., 2011; Baturay and Toker, 2015; Alharthi et al., 2019). For example, Vitak et al. (2011) found a significant relationship between age and cyberloafing among male subjects, indicating that younger men participate more in cyberloafing behaviors. Baturay and Toker (2015) found that compared with other demographic variables such as age, gender has the greatest impact on cyberloafing and that males are more likely to engage in cyberloafing.
2. Personality traits include extraversion, agreeableness, neuroticism, conscientiousness, and openness (Jia et al., 2013; Kim et al., 2016; Yildiz Durak and Saritepeci, 2019). Extraversion, for example, was found to have a positive relationship with cyberloafing. Employees with high levels of extraversion seek to interact more with others, thereby exhibiting more online behaviors to enhance their social strength and build career networks (Jia et al., 2013).
3. Individual cognition, such as perceived overqualification (Cheng et al., 2020; Zhang J. et al., 2020). It is found that when employees with perceived overqualification failed to achieve their need for achievement, they tend to feel more frustrated and are less likely to experience harmonious passion at work, ultimately resulting in cyberloafing (Cheng et al., 2020). Similarly, the studies of Zhang J. et al. (2020) and other researchers have found that employees with perceived overqualification believe their qualifications exceed job requirements and are more likely to have moral disengagement and anger toward the organization, resulting in cyberloafing.
4. Job characteristics, including meaningful work, workload, and job demands (Askew et al., 2014; Usman et al., 2021; Hensel and Kacprzak, 2020). Meaningful work inhibits cyberloafing through employees’ affective commitment, and leader-member exchange moderates this process (Usman et al., 2021). On the other hand, Hensel and Kacprzak (2020) found that job overload affects employees’ cyberloafing behavior.
5. Situational factors, including workplace ostracism and abusive supervision (Koay, 2018; Yeik et al., 2018; Agarwal and Avey, 2020; Lim et al., 2021), and others. For example, Koay (2018) found the positive effect of workplace ostracism on emotional exhaustion, which in turn affects cyberloafing, with facilitating conditions moderating this process. Similarly, Agarwal and Avey (2020) found that abusive supervision can reduce the psychological capital of employees, which in turn leads to employee cyberloafing, with psychological contract breaches playing a borderline role.

The review of the literature reveals that scholars paid little attention to the relationship between leadership style and cyberloafing (Tandon et al., 2022). Thus, we propose to examine the influence of authoritarian leadership on employee cyberloafing and its underlying mechanism.

### Authoritarian leadership and cyberloafing

Authoritarian leaders emphasize authority and demand absolute obedience from subordinates. Authoritarian leadership includes four specific types of behaviors: (1) didactic style, (2) belittling subordinates, (3) image decoration, and (4) lecturing (Pellegrini and Scandura, 2008). First, the didactic style is manifested when the leader holds the power, makes his own decisions in everything, prefers downward communication, and closely monitors subordinates. Second, belittling subordinates refers to the leader’s intentional disregard for the suggestions and contributions of subordinates. Third, image decoration means that leaders manipulate information deliberately to maintain their authority. Fourth, lecturing is the leaders’ behavior of reprimanding and directing underperforming subordinates. Many researchers have studied the impact of authoritarian leadership and found that authoritarian leadership has a significant negative impact on subordinate creativity, job performance, intention to stay, affective commitment, and organizational citizenship behavior (Schaubroeck et al., 2017; Zhang and Xie, 2017; Guo et al., 2018), and a significant positive impact on subordinate resistance behavior, silence behavior, and workplace deviance (Duan et al., 2017; Zhang Y. et al., 2020; Zheng et al., 2020).

Indeed, authoritarian leadership plays a more non-functional role in the attitudes and behaviors of subordinates, which may also be true for cyberloafing. First, authoritarian leadership, as a major source of stress, raises employees’ perceptions of work stress and reduces psychological empowerment. Employees’ self-control resources are depleted when they intentionally cope with stress. Second, since authoritarian leaders require subordinates to obey orders without questioning existing procedures, employees are deprived of the opportunity to participate in decision-making and engage in active communication with leaders, which not only dampens their enthusiasm for high performance but also deepens their concern about future career development, and constantly consumes their cognitive resources. Third, authoritarian bosses undervalue subordinates, reprimand underperforming employees, and disregard subordinates’ contributions (Guo et al., 2018).

Employees working for authoritarian leaders frequently experience a reduction in self-efficacy and the development of negative emotions, culminating in the depletion of emotional resources. Finally, authoritarian bosses prioritize centralization and withhold comprehensive work-related information from employees. Inadequately informed, employees will inevitably spend more time and energy on collecting work data, leading to insufficient physiological resources. Individuals will adopt resource acquisition behaviors in a response to resource depletion, following the notion of resource conservation. For example, during working hours, employees will converse with and confide in others in the virtual world to satisfy their emotional and cognitive resource demands (Yan et al., 2020). They will also play games or shop online to increase physical resources (She and Li, 2022). Or they will boost their regulatory resources by watching short videos and having other micro-breaks (Syrek et al., 2018). Based on the above review, it is argued that authoritarian leadership encourages subordinates’ cyberloafing behavior. Therefore, we suggest the following hypothesis:

*Hypothesis 1*: Authoritarian leadership positively affects the cyberloafing behavior of subordinates.

### The mediating role of emotional exhaustion

Conservation of resource theory states that individuals tend to acquire, maintain, nurture and protect their cherished resources (Hobfoll, 1989, 2001). People typically use resources to deal with stress in the present circumstances, as well as to invest in resources and to gain new resources. When an individual’s resource is spent or the expected return on resource investment is not realized, he or she experiences emotional exhaustion (Wright and Cropanzano, 1998; Hobfoll, 2001). Emotional exhaustion is the core dimension of burnout, which refers to the psychological exhaustion of employees after a large amount of emotional and physiological resources are consumed. Previous studies have shown that emotional exhaustion serves as a link between stressful events and employee behavior. For instance, emotional exhaustion has been found to mediate the relationship between workplace ostracism and unethical behavior (Qi et al., 2020), as well as the relationship between the sources of stress and work-family balance (Ma et al., 2021).

Authoritarian leadership is viewed as a source of job stress in the conservation of resource theory. Employees experience actual resource loss and perceive a threat to their valued resources when confronted with this impediment stressor, and they must use additional effort to deal with the stress. First, authoritarian leaders keep strict control of subordinates and absolute dominance in the organization by controlling and manipulating information. Employees can only obey orders from leaders since they lack comprehension of organizational plans and information on job assignments. Since employees need to devote additional physical and psychological resources to seek and confirm job information, a state of emotional exhaustion is more likely to occur. Second, authoritarian leaders advocate an autocratic management style. Employees lose excitement and initiative at work because they are not allowed to participate in decision-making.

Employees working under authoritarian leader lack career development plans and growth opportunities, which increases their experience of insecurity at work, reduces their sense of accomplishment and increases their resource consumption. Employees can only use their resources to adjust their work status, and no longer have enough resources to face the stressors in the organizational situation, resulting in emotional exhaustion. Third, authoritarian leaders would deliberately disregard the subordinates’ suggestions and contributions while attributing failures to them. Employees’ self-esteem and self-efficacy will be undermined, resulting in negative emotions and the consumption of employees’ emotional resources. Fourth, authoritarian leadership expects superior performance from subordinates and aggressively penalizes underperformers. Employees’ problems at work or in life cannot be solved promptly because authoritarian leaders neglect their difficulties. Due to the characteristics of authoritarian leadership above, employees will find it impossible to replenish their limited resources and end up in a breakdown resulting in emotional exhaustion.

The conservation of resource theory suggests that when people sense a decline in their resources, they tend to safeguard their resources and are driven to compensate for the lost resources (Hobfoll, 1998, 2001). Emotionally exhausted employees will lessen their work commitment, reduce their workload, or take other measures to protect, maintain, and acquire emotional resources (Anwar et al., 2020). Individuals who indulge in cyberloafing can temporarily detach emotionally exhausted employees from work, assist them in escape from stress and negativity, and enable employees to take a defensive attitude to protect or even replenish their resources such as cognitive and regulatory resources through relaxation. For example, when employees are emotionally exhausted, they can protest their displeasure with the leader by communicating with others online, and they can also divert their attention by browsing online news.

In addition, as employees develop emotional exhaustion and dissatisfaction, cyberloafing is taken as a way to retaliate against leaders and organizations. Prior studies found that cyberloafing has several negative implications, including decreased job performance and mental health impairment (Wu et al., 2020a; She and Li, 2022). There is an old Chinese proverb, “*Excessive attention to a plaything saps the will.*” When employees slack off online to escape from the pressure and misery at work, they separate themselves from leaders and diminish their work efficiency and quality, resulting in a vicious spiral of cyberloafing. In sum, emotionally exhausted employees are more likely to engage in cyberloafing to cope with stress and vent their emotions. Authoritarian leadership can cause emotional exhaustion in subordinates, which leads to cyberloafing as a coping mechanism. Therefore, the following hypothesis is proposed:

*Hypothesis 2*: Emotional exhaustion plays a mediating role between authoritarian leadership and subordinate cyberloafing.

### The moderating role of power distance orientation

The degree to which individuals accept the unequal distribution of power in organizations or institutions is referred to as power distance orientation (Kirkman et al., 2009). Power distance orientation was originally defined at the national level as the extent to which a society accepts the unequal distribution of power within an organization or institution. But researchers have discovered that this value dimension varies widely between individuals, and can affect many outcomes. Some studies, for example, have found that power distance orientation has a negative moderating effect on the relationship between abusive supervision and emotional exhaustion (Vesa, 2018). Similarly, Yan et al. (2020) have found that power distance orientation has a negative moderating effect on the relationship between ingratiation and counterproductive behavior.

According to the conservation of resource theory, individuals respond to stressors and evaluate resources differently (Hobfoll, 1998). As a typical individual trait, power distance orientation represents an individual’s attitude toward the unequal power distribution (Farh et al., 2007). Studies have shown that the level of employees’ power distance orientation reflects their level of identification with leadership authority, which may lead to their different attitudes toward leaders’ negative behavior (Wang et al., 2019; Yan et al., 2020). Employees with a high-power distance orientation accept authoritarian leaders’ top-down communication style, respect authority, and hierarchy, and recognize leaders’ superiority. Consequently, they are more willing to accept leaders’ opinions, follow orders (Javidan et al., 2006), and rarely question leaders’ decisions and instructions. These employees tolerate leaders’ oversight and authority, and consider them as the given right of leaders. Some scholars suggested that employees with high power distance orientation are accustomed to the authority of the leader and often rely on the leadership to make decisions. Autocratic decision-making by leaders can even boost employee job satisfaction (Yan et al., 2020). In this case, employees do not need to devote extra resources and efforts to autonomous work and decision-making, which protects their resources to a certain extent, and is less likely to cause emotional exhaustion among employees.

On the other hand, employees with a low power distance orientation, advocate for equality between leader and employee. They believe that everyone has the right to free speech and that employees and leaders are solely distinguished by their work responsibilities. These employees do not accept the superiority of leaders in social status and refuse to take unreasonable orders from them (Chen and Aryee, 2007). They prefer consultative leaders and are more likely to develop close relationships with them. They are willing to offer insights and advice to the organization and dare to challenge the viewpoints of high-ranking individuals (Kirkman et al., 2009). Furthermore, failure to create an equal relationship with their superiors will reduce their identification with the organization and deplete their resources, making them more sensitive to resource loss and more vulnerable to emotional exhaustion due to mental stress. Employees with different power distance orientations can have different perceptions of authoritarian leadership, resulting in different emotions and attitudes. Therefore, we suggest the following hypothesis:

*Hypothesis 3*: Power distance orientation negatively moderates the relationship between authoritarian leadership and subordinate emotional exhaustion. When power distance orientation is low, authoritarian leadership has a stronger positive effect on employee emotional exhaustion.

In Hypothesis 2, we propose that emotional exhaustion mediates the relationship between authoritarian leadership and cyberloafing. Combined with the analysis of Hypothesis 3, power distance orientation moderates the relationship between authoritarian leadership and emotional exhaustion. When employees have relatively higher power distance orientation, they may be more obedient to authoritarian leaders’ orders, more tolerant of their behavior, more accustomed to the leader’s supervision, criticism, and information manipulation, and less likely to experience emotional exhaustion and engage in cyberloafing. Therefore, we argue that high power distance orientation will moderate the cyberloafing behaviors induced by authoritarian leaders through employee emotional exhaustion. On the contrary, employees with a low power distance orientation will question leaders’ decision and tend to have more disagreements in ideas and behaviors with the leader, resulting in the necessity to engage in cyberloafing to defend and replenish resources. Based on the analysis above, it is argued that low power distance orientation will exacerbate the emotional exhaustion caused by authoritarian leadership and increase the frequency of cyberloafing behavior. Therefore, we suggest the following hypothesis:

*Hypothesis 4*: Power distance orientation negatively moderates the indirect effect of authoritarian leadership on subordinate cyberloafing via emotional exhaustion. This indirect effect is enhanced when the power distance orientation is low.

**Figure 1:**
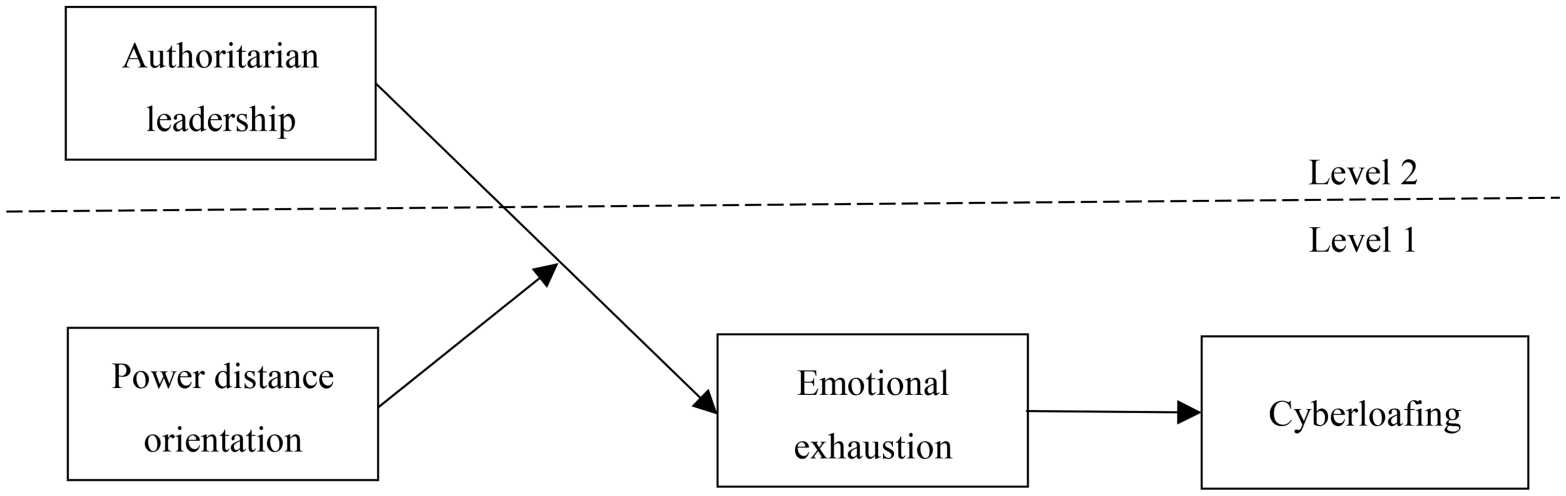
Theoretical framework.

To sum up, drawing upon the conservation of resource theory, this study attempts to explore the mechanism of authoritarian leadership affecting subordinate cyberloafing through the mediating role of subordinate emotional exhaustion and the moderating role of power distance orientation. The theoretical model of this paper is shown in Figure 1.

## Materials and methods

### Participants and procedures

Data were collected in three steps by utilizing paper-based survey questionnaires distributed among corporate employees from companies located in Guizhou Province, China, covering the industries of finance, retail, and service. First, the researchers informed the heads of human resources of each organization about the purpose of the study and invited them to randomly select teams to participate in the survey. Second, researchers coded team members who participated in the study and put the instructions and questionnaires into coded envelopes. Finally, researchers distributed the questionnaires on site, and the questionnaires were sealed by the participants and collected by the researchers on the spot.

In order to motivate our survey participants and increase the validity of the responses, each participant was given a small gift after they filled out the survey questionnaires. To reduce the effect of common method bias, the data were collected at two time points. At time point 1, we invited employees to fill in demographic factors, authoritarian leadership, and power distance orientation. A total of 420 questionnaires were distributed and 396 questionnaires were retrieved. At time point 2, we asked employees who completed the survey at T1 to report on emotional exhaustion and cyberloafing. A total of 396 copies were distributed and 378 copies were returned.

After removing feedback with random responses, missing data, and unmatched time points, paired data of 360 employees working in 103 groups were collected. The gender of participants in the final sample pool was predominantly male (233 respondents = 64.72%), the largest age group was 26–35 years old (140 respondents = 38.89%), and more than half received undergraduate education (203 respondents = 56.39%), and their working time with leaders was primarily 7–12 months (116 respondents = 32.22%).

### Measures

Authoritarian leadership, emotional exhaustion, power distance orientation, cyberloafing, and leader-member exchange were measured using a 5-point Likert-type scale.

#### Authoritarian leadership

The five-item scale developed by Zhang et al. (2011) was used to measure authoritarian leadership. A sample item was, “*My supervisor has personal control of most matters*.” The Cronbach’s alpha coefficient of the scale was 0.97. The intra-class correlation coefficients ICC(1), ICC(2), and the mean *RWG* (within-group interrater reliability) values of authoritarian leaders were 0.57, 0.82, and 0.98 respectively, meeting the aggregation requirements.

#### Emotional exhaustion

Emotional exhaustion was measured by the three-item scale developed by Watkins et al. (2014). A sample item was, *“I feel burned out from my work.”* The Cronbach’s alpha coefficient of the scale was 0.90.

#### Power distance orientation

Power distance orientation was measured by the six-item scale developed by Dorfman and Howell (1988). A sample item was, “*Supervisors should make most decisions without consulting subordinates*.” The Cronbach’s alpha coefficient of the scale in this sample was 0.96.

#### Cyberloafing

The three-item scale developed by Lim et al. (2021) was used to measure cyberloafing. A sample item was, *“I access the Internet at work for non-work-related purposes several times each day.”* The Cronbach’s Alpha coefficient of the scale was 0.93.

Based on Zhang Y. et al. (2020) and Agarwal and Avey (2020), we controlled for gender, age, education, and tenure with a leader. Leader-member exchange (LMX) is often used by scholars to account for the mediating mechanism through which authoritarian leadership influences subordinates’ cognition, attitude, and behavior (Gu et al., 2018; Nazir et al., 2021). To prove that emotional exhaustion is a new mediating variable, we also control for leader-member exchange. The leader-member exchange adopts the scale developed by Graen and Uhl-Bien (1995), which contains 7 items. A sample item was, *“My supervisor would be personally inclined to use his or her power to help me solve problems in my work.”* The Cronbach’s alpha coefficient of the scale was 0.94.

## Results

### Confirmatory factor analysis

We used AMOS v.26 to conduct confirmatory factor analysis (CFA) to test the discriminant validity of authoritarian leadership, emotional exhaustion, power distance orientation, cyberloafing, and leader-member exchange (please see Table 1 for CFA results). The results showed that the five-factor model (*χ*^2^/*df* = 2.02, CFI = 0.97, TLI = 0.97, RMSEA = 0.05, SRMR = 0.03) was significantly better than other nested models, indicating that the five variables had good discriminant validity.

**Table 1:** Results of confirmatory factor analysis.

Model	*χ^2^*	*df*	*χ^2^/df*	*∆χ^2^(∆df)*	CFI	TLI	RMSEA	SRMR
Five-factor model	488.05	242	2.02		0.97	0.97	0.05	0.03
Four-factor model	1278.78	246	5.20	790.73^***^(4)	0.89	0.88	0.11	0.14
Three-factor model	2149.80	249	8.63	1661.75^***^(7)	0.80	0.77	0.15	0.16
Two-factor model	4302.45	251	17.14	3814.40^***^(9)	0.57	0.52	0.21	0.24
One-factor model	6582.38	252	26.12	6094.33^***^(10)	0.32	0.26	0.27	0.26

*N* (Level 2) = 103; *N* (Level 1) = 360; Four-factor model, Combining emotional exhaustion and power distance orientation into one factor; Three-factor model, Combining emotional exhaustion and power distance orientation into one factor, and combining authoritarian leadership and cyberloafing into one factor; Two-factor model, Combining emotional exhaustion and power distance orientation into one factor, and combining authoritarian leadership, cyberloafing and leader-member exchange into one factor; One-factor model, Combining all constructs into a factor.

*** *p* < 0.001.

### Descriptive statistics analysis

We utilized SPSS v.26 to conduct descriptive statistical analysis. The mean, standard deviation, and correlation coefficient of each variable are shown in Table 2. The results show that emotional exhaustion is positively correlated with cyberloafing (*r* = 0.49, *p* < 0.01).

**Table 2:** Results of descriptive statistical analysis.

	*M*	*SD*	1	2	3	4	5	6	7
**Level 1**									
1. Sex	0.65	0.48							
2. Age	2.46	0.97	−0.09						
3. Education level	2.79	0.83	0.08	−0.09					
4. Tenure with leader	2.19	1.04	0.12^*^	0.01	−0.04				
5. Leader-member exchange	3.68	0.72	0.04	0.02	−0.04	−0.02			
6. Emotional exhaustion	3.98	0.81	−0.14^*^	0.13^*^	0.04	0.03	−0.01		
7. Power distance orientation	3.02	1.14	0.00	−0.06	−0.03	−0.10	0.03	0.20^**^	
8. Cyberloafing	4.27	0.77	−0.10	0.02	0.10	−0.02	−0.06	0.49^**^	0.10
**Level 2**									
1. Authoritarian leadership	4.67	0.67							

*N* (Level 2) = 103; *N* (Level 1) = 360; Sex: Male (0), female (1); Age: ≤25 years (1), 26–35 years (2), 36–45 years (3), ≥46 years (4); Education level: Junior college (1), bachelor degree (2), graduate degree (3); Tenure with leader: ≤6 months (1), 7–12 months (2), 13–24 months (3), ≥25 months (4).

* *p* < 0.05;

** *p* < 0.01.

### Hypotheses testing

To test the hypotheses, we used HLM 8.1 to conduct a multilevel analysis (the results are shown in Table 3). Hypothesis 1 suggested that authoritarian leadership positively influences the cyberloafing behavior of subordinates. To test H1, gender, age, education level, tenure with a leader, leader-member exchange, and authoritarian leadership were simultaneously entered into the regression equation with cyberloafing as the dependent variable. According to Model 4, authoritarian leadership has a significant positive impact on employee cyberloafing (*β* = 0.49, *p* < 0.001). Thus, H1 was supported.

**Table 3:** Results of hierarchical linear modeling.

	Emotional exhaustion		Cyberloafing		
	Model 1	Model 2	Model 3	Model 4	Model 5
Intercept	3.96^***^(0.04)	3.99^***^(0.04)	4.25^***^(0.05)	4.24^***^(0.04)	4.25^***^(0.04)
**Level 1**					
Sex	−0.16^*^(0.08)	−0.18^*^(0.07)	−0.13(0.07)	−0.10(0.07)	−0.03(0.06)
Age	0.08^*^(0.04)	0.08^*^(0.04)	0.03(0.04)	0.01(0.04)	−0.01(0.04)
Education level	−0.01(0.04)	−0.03(0.05)	0.06(0.06)	0.03(0.04)	0.04(0.05)
Tenure with leader	0.05(0.04)	0.05(0.03)	0.01(0.04)	0.01(0.04)	−0.01(0.03)
Leader-member exchange	0.03(0.05)	0.01(0.05)	−0.05(0.07)	−0.03(0.07)	−0.04(0.06)
Emotional exhaustion					0.39^***^(0.05)
Power distance orientation		0.07^*^(0.03)			
**Level 2**					
Authoritarian Leadership	0.61^***^(0.09)	0.43^***^(0.09)		0.49^***^(0.10)	0.23^*^(0.09)
**Interaction**					
Authoritarian leadership × power distance orientation		−0.18^**^(0.06)			
∆ R^2^	0.26	0.33	0.12	0.28	0.41

*N* (Level 2) = 103; *N* (Level 1) = 360.

* *p* < 0.05;

** *p* < 0.01;

*** *p* < 0.001.

Hypothesis 2 suggested that emotional exhaustion would mediate the relationship between authoritarian leadership and cyberloafing. To test H2, gender, age, education level, tenure with a leader, leader-member exchange, authoritarian leadership, and emotional exhaustion were simultaneously entered into the regression equation with cyberloafing as the dependent variable. According to Model 5, emotional exhaustion positively affects cyberloafing (*β* = 0.39, *p* < 0.001), but the positive effect of authoritarian leadership on employee cyberloafing is significantly reduced (*β* = 0.23, *p* < 0.05). This suggests that emotional exhaustion mediates the relationship between authoritarian leadership and employee cyberloafing. Hence, H2 received initial support.

We used R software to conduct the Bootstrapping test on the significance of the mediating effect of emotional exhaustion between authoritarian leadership and employee cyberloafing (Preacher and Selig, 2012). The results showed that the mediating effect of authoritarian leadership on employee cyberloafing *via* emotional exhaustion was 0.24, with a 95% confidence interval (*CI*) of [0.15, 0.34], excluding 0. It can be seen that the mediating effect of emotional exhaustion was significant. Thus, H2 was fully supported.

**Figure 2:**
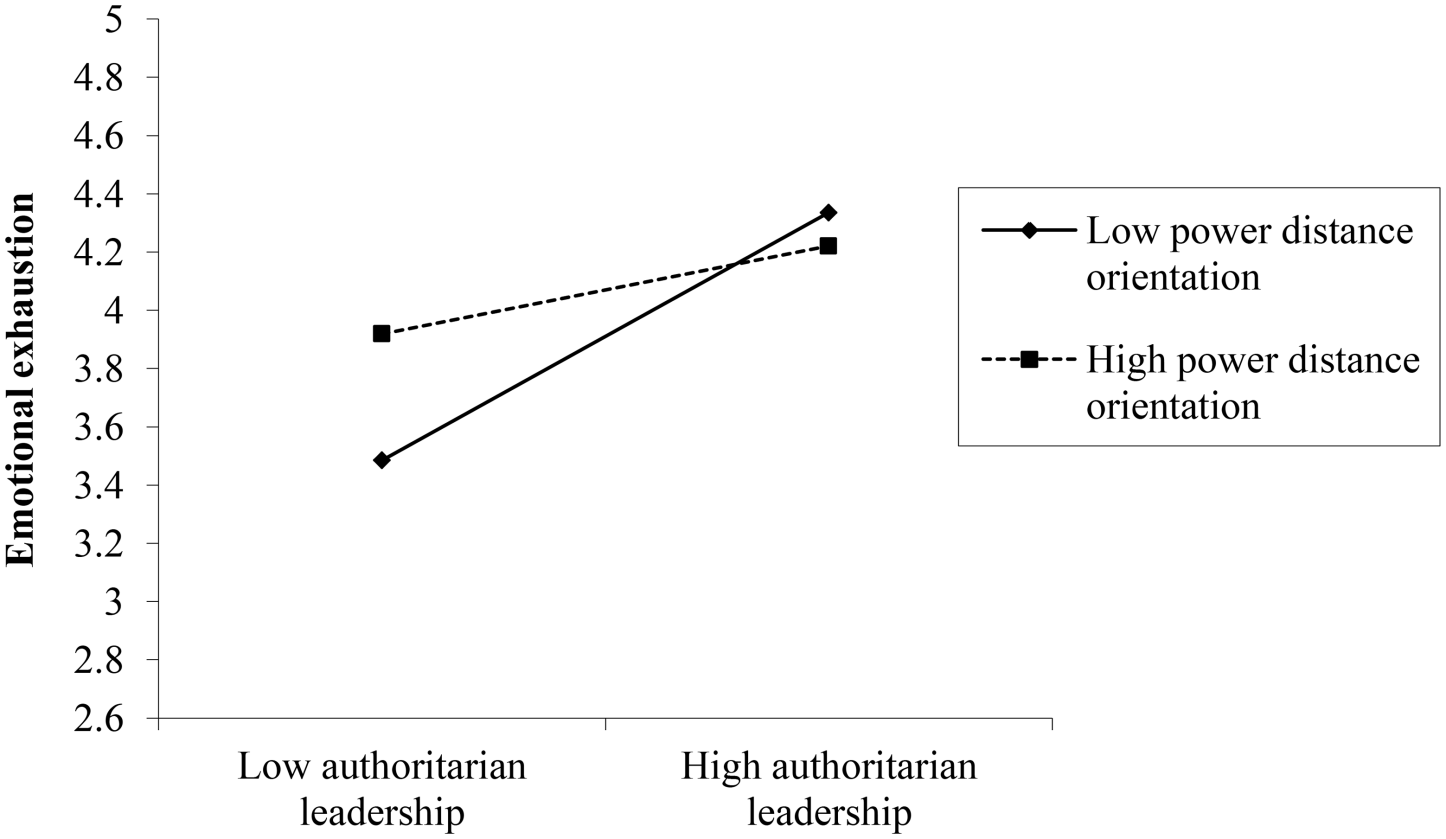
The moderating effect of power distance orientation on the relationship between authoritarian leadership and emotional exhaustion.

Hypothesis 3 proposed that power distance orientation would negatively moderate the relationship between authoritarian leadership and subordinate emotional exhaustion. To test H3, gender, age, education level, tenure with a leader, leader-member exchange, authoritarian leadership, power distance orientation, and their interaction terms were simultaneously entered into the regression equation with emotional exhaustion as the dependent variable. According to Model 2, the interaction term between authoritarian leadership and power distance orientation has a significant negative impact on emotional exhaustion (*β* = −0.18, *p* < 0.01), which revealed that power distance orientation negatively moderated the relationship between authoritarian leadership and emotional exhaustion. Furthermore, Figure 2 portrays the moderating effect of power distance orientation. At the same time, the results of a simple slope analysis showed that the higher the employee power distance orientation, the weaker the positive association between authoritarian leadership and emotional exhaustion (*β* = 0.23, *p* < 0.05), and the lower the employee power distance orientation, the stronger the positive association between authoritarian leadership and emotional exhaustion (*β* = 0.64, *p* < 0.001). Thus, H3 received initial support.

As recommended by Preacher and Selig (2012), we used R software to conduct the Bootstrapping test on the significance of moderated mediation effect. The results showed (see Table 4) that the positive association between authoritarian leadership and emotional exhaustion was significant at both high (*β* = 0.23, 95% *CI* = [0.01, 0.45]) and low (*β* = 0.64, 95% *CI* = [0.41, 0.86]) power distance orientation levels, and the difference (*β* = −0.41, 95% *CI* = [−0.68, −0.14]) was also significant. Thus, H3 was fully supported. Table 4 revealed that the indirect effect of emotional exhaustion on the relationship between authoritarian leadership and cyberloafing was significant at both high (*β* = 0.08, 95% *CI* = [0.001, 0.17]) and low (*β* = 0.23, 95% *CI* = [0.14, 0.34]) power distance orientation levels, and the difference (*β* = −0.15, 95% *CI* = [−0.26, −0.05]) was also significant, indicating that power distance orientation moderated the indirect effect of authoritarian leadership on cyberloafing through emotional exhaustion. As a result, H4 was supported.

**Table 4:** Results of moderated mediation analysis.

	Moderator	Authoritarian leadership → emotional exhaustion	Direct effect	Indirect effect	Total effect
Cyberloafing	High power distance orientation	0.23^*^ [0.01, 0.45]	−0.001 [−0.21, 0.21]	0.08^*^ [0.001, 0.17]	0.08 [−0.15, 0.30]
	Low power distance orientation	0.64^*^ [0.41, 0.86]	0.34^*^ [0.13, 0.55]	0.23^*^ [0.14, 0.34]	0.57^*^ [0.34, 0.81]
	Differences (*Δ*)	−0.41^*^ [−0.68, −0.14]	−0.34^*^ [−0.57, −0.12]	−0.15^*^ [−0.26, −0.05]	−0.49^*^ [−0.74, −0.24]

*N* (Level 2) = 103; *N* (Level 1) = 360; 95% confidence interval in parentheses.*p < 0.05.

## Discussion

The internet has accelerated global informatization while also creating new prospects for societal growth. In today’s business setting, employees’ dependency on the Internet leads to the prevalent phenomenon of cyberloafing. What is worse, authoritarian leadership can lead to emotional exhaustion in employees, exacerbating their cyberloafing behavior. Thus, investigating how authoritarian leadership affects employees’ emotional exhaustion and cyberloafing has both theoretical and practical implications.

### Theoretical significance

Drawing upon the conservation of resource theory, we examined the process mechanism and boundary conditions of authoritarian leadership affecting subordinate cyberloafing. The findings revealed that authoritarian leadership causes subordinate cyberloafing *via* emotional exhaustion. Moreover, employee power distance orientation not only negatively moderates the relationship between authoritarian leadership and subordinate cyberloafing, but also negatively moderates the mediating role of emotional exhaustion in the relationship between authoritarian leadership and subordinate cyberloafing. Our findings shed light on the understanding of authoritarian leadership and cyberloafing in the following respects:

First, our study expanded the research on the antecedents of cyberloafing and bridged the research gap between authoritarian leadership and subordinate cyberloafing. Although a series of insightful studies have been conducted on the antecedents of cyberloafing in recent years, these studies are limited to demographics, personality traits, individual cognition, job characteristics, and situational factors. For example, gender, Big-Five personality, perceived overqualification, and job demands can all effectively predict employees’ cyberloafing behavior. Similarly, some scholars have examined the impact of leadership styles on cyberloafing, such as abusive supervision (Agarwal and Avey, 2020) and leader mindfulness (Zoghbi-Manrique-De-Lara et al., 2020), but there is still a lack of attention to the multiple leadership styles, particularly the authoritarian leadership prevalent in Chinese organizational context.

Furthermore, prior studies primarily focused on authoritarian leadership and its impact on ethical voice (Zheng et al., 2019), workplace deviance (Zheng et al., 2020), silence behavior (Duan et al., 2017), and others. Entering the era of “*Internet +*,” cyberloafing has become a non-negligible behavior that has a significant impact on organizational performance (She and Li, 2022). Therefore, it is imperative to study the mechanism by which authoritarian leadership influences cyberloafing. We examined the positive effect of authoritarian leadership on subordinates’ cyberloafing, which has not only expanded the research on the antecedents of cyberloafing but also responded to the call for research on the effects of authoritarian leadership (Gu et al., 2018).

In addition, we examined the process mechanism of authoritarian leadership affecting subordinate cyberloafing, and revealed the role of employee resource depletion in this association. Recent studies drew on the job demands-resources model (Elrehail et al., 2021), affective event theory (Hu et al., 2021), social exchange theory (Raza and Ahmed, 2020) and equity theory (Cheng et al., 2020), and other theoretical perspectives to explore the relationship between cyberloafing and other variables. However, these studies neglected the loss, protection, and acquisition of resources at the individual level. Hence, drawing upon the theory of resource conservation, we examined the resource operation mechanism of authoritarian leadership inducing subordinates’ cyberloafing behavior. The findings respond to the researchers’ call to investigate the antecedent mechanism of cyberloafing, broaden the scope of previous studies on the mediators of other variables affecting cyberloafing, including work pressure (Elrehail et al., 2021), workplace loneliness (Hu et al., 2021), psychological capital (Raza and Ahmed, 2020), and harmonious passion (Cheng et al., 2020), and extend the application of resource conservation theory.

Finally, we also tested the moderating role of power distance orientation in the process mechanism of authoritarian leadership affecting subordinate cyberloafing through emotional exhaustion, and it further defines the impact of authoritarian leadership. Our study not only sheds light on the negative effects of authoritarian leadership but also on the boundary conditions under which these effects intensify and weaken. Previous studies have explored the moderators of the relationship between authoritarian leadership, including employee cognition, attitude, and behavior, psychological capital (Guo et al., 2018), dependence on a leader (Wang et al., 2019), trust in leadership (Du et al., 2019), and benevolent leadership (Zheng et al., 2019), etc., but neglected the role of traditional culture in personal characteristics. Based on resource conservation theory, we argued and confirmed that power distance orientation negatively moderated the relationship between authoritarian leadership and emotional exhaustion, as well as the indirect effect of authoritarian leadership on subordinate cyberloafing through emotional exhaustion. This finding expands our knowledge of the relationship between authoritarian leadership and subordinate cyberloafing.

### Practical significance

Our study demonstrated that authoritarian leadership triggers subordinates’ cyberloafing behavior. First of all, the organization should select managers who have both managerial competence and moral integrity. Managers should be provided with frequent training to improve their flexibility and emotional control in managerial practice. And managers should not only keep a certain power distance orientation from their subordinates, but they should also build a good relationship with their subordinates to acquire their trust. Furthermore, while managers must maintain their rights in decision-making and control, they should also give reasonable discretion to subordinates to build employee self-esteem and create a fair and inclusive organizational atmosphere, which can effectively prevent cyberloafing in the workplace and empower employees to contribute more actively to the development of the organization. Second, supervisors should be aware of the potential consequences of workplace cyberloafing. Employees’ online behavior must be guided during working hours to reduce cyberloafing behavior, and a cyber security system must be in place to educate employees on the appropriate standards of online behavior.

Third, we investigated the mechanism of emotional exhaustion in the process of authoritarian leadership affecting subordinate cyberloafing. We contend that the organization should follow the “people-oriented” management philosophy. Managers must provide staff with psychological resources such as respect, affirmation, and support in addition to material resources to fulfill tasks. Regular training, a counseling room, and a venting room are some of the measures to improve employees’ emotional guidance and stress coping skills and to allow employees to experience intense emotions to vent in a timely and effective manner. These measures are conducive to the development of employees and enterprises through the expansion of employees’ resource-gathering channels and the lowering of their emotional exhaustion level. Fourth, organizations should create additional channels of communication for organizational members to voice their opinions and difficulties, so that managers can provide timely assistance to reduce employees’ mental fatigue. Fifth, organizations should manage staff workload and working hours to prevent employee resource depletion. Allowing proper leisure and enjoyment at work can also aid in the replenishment of resources and the avoidance of emotional exhaustion.

Finally, we found that power distance orientation negatively moderated the relationship between authoritarian leadership and emotional exhaustion. This impact implies that organizations should manage individuals differently depending on their power distance orientation. To begin, questionnaire evaluation can be used in employee recruitment and training to determine different employees’ power distance orientation levels and to achieve better leadership-employee matches during team building. For instance, authoritarian leaders need to be matched with employees with low power distance orientation, and vice versa. Managers must be able to switch leadership styles and managerial tactics according to the varying levels of power distance orientation among team members in order to achieve organizational goals. For instance, when dealing with employees with low power distance orientation, managers might use a democratic approach to assign duties based on employees’ choices, so that their work motivation, commitment, and efficiency can be improved.

## Limitations and future prospects

The following are the limitations of this study. First, because the survey data in this study is limited to Chinese enterprises, the generalizability and universality of the research findings may be constrained. To further examine the findings of this study, future research may try choosing samples from other cultural backgrounds.

Second, exclusively from the perspective of the conservation of resource theory, we examined the function of emotional exhaustion as the mediating mechanism in the relationship between authoritarian leadership and employee cyberloafing. Future researchers may apply other theoretical approaches, e.g., affective event theory, social exchange theory, and social identity theory to investigate whether authoritarian leadership causes employees cyberloafing behavior. For example, based on affective event theory, authoritarian leaders may induce employees’ cyberloafing behaviors by stimulating employees’ anger or anxiety. Also, based on the theory of social identity, authoritarian leadership may reduce employees’ sense of identification with the organization and leadership, and engage in cyberloafing. Therefore, we invite future researchers to examine the impact of authoritarian leadership on employee cyberloafing from a novel theoretical perspective, as well as to enhance its internal transmission mechanism.

Third, we looked at the moderating role of employee power distance orientation in the process of authoritarian leadership affecting employee emotional exhaustion and cyberloafing. First of all, in addition to power distance orientation, there are other individual differences or situational factors such as political skills and psychological rights. For example, employees with stronger political skills have better relationship networks in the organization, get along more naturally with leaders, understand and adjust to the style of leaders better, and adopt positive coping strategies to reduce negative behaviors. Secondly, the occurrences of employee behavior vary with not only individual differences but also environmental factors such as organizational justice or benevolent leadership. For example, when employees perceive organizational unfairness, they will be more sensitive to leadership authority, which is more likely to cause emotional exhaustion. As a result, we recommend further examination of the potential boundary conditions of authoritarian leadership and cyberloafing.

Finally, we collected data for the mediating and the outcome variables at the same time, which may not explain the causal relationship between the two. To better determine the causal relationship between variables, future research may consider collecting data at three different time points for each category of variables in the research model, i.e., predictor, mediator, and outcome variables. In addition, cyberloafing is concealed in nature and difficult to observe (Agarwal and Avey, 2020). Since this study collected all the data from a single source, common method bias is indeed one limitation of this paper. Future research can try to invite colleagues to evaluate employees’ cyberloafing to solve this problem.

## Conclusion

Drawing upon the conservation of resource theory, we found that authoritarian leadership influences subordinate cyberloafing behavior which is further contingent upon emotional exhaustion and the differing levels of power distance orientation. We tested the main effect of the relationship between authoritative leadership and cyberloafing *via* the mediating mechanism of emotional exhaustion and the varying levels of power distance orientation. Our study offers new directions and insights as well as future recommendations for research on negative leadership styles and employee cyberloafing.

## Data availability statement

The original contributions presented in the study are included in the article/supplementary material; further inquiries can be directed to the corresponding author.

## Ethics statement

Ethical approval was not provided for this study on human participants because an ethics approval was not required as per our institution’s guidelines and national regulations. The patients/ participants provided their written informed consent to participate in this study.

## Author contributions

YZ: conceptualization, data curation, and writing—original draft. JW: writing—original draft. MA: writing and editing. YW: data analysis, writing and editing. All authors contributed to the article and approved the submitted version.

## Funding

We acknowledge the financial support from the National Social Science Foundation of China (19BGL112).

## Conflict of interest

The authors declare that the research was conducted in the absence of any commercial or financial relationships that could be construed as a potential conflict of interest.

## Publisher’s note

All claims expressed in this article are solely those of the authors and do not necessarily represent those of their affiliated organizations, or those of the publisher, the editors and the reviewers. Any product that may be evaluated in this article, or claim that may be made by its manufacturer, is not guaranteed or endorsed by the publisher.
